# Adolescent depressive disorders and family based interventions in the family options multicenter evaluation: study protocol for a randomized controlled trial

**DOI:** 10.1186/1745-6215-14-384

**Published:** 2013-11-13

**Authors:** Andrew J Lewis, Melanie D Bertino, Joanna Skewes, Lyndel Shand, Nina Borojevic, Tess Knight, Dan I Lubman, John W Toumbourou

**Affiliations:** 1 School of Psychology, Faculty of Health, Deakin University, Burwood Highway, Burwood, Victoria 3125, Australia; 2 Eastern Health Clinical School, Monash University, Melbourne, Australia; 3 Turning Point Alcohol and Drug Center, Eastern Health, Melbourne, Australia; 4 Center for Mental Health and Wellbeing Research, Deakin University, Burwood Highway, Burwood, Victoria 3125, Australia

**Keywords:** Depression, Adolescents, Family-based interventions, Youth mental health, Randomised clinical trial

## Abstract

**Background:**

There is increasing community and government recognition of the magnitude and impact of adolescent depression. Family based interventions have significant potential to address known risk factors for adolescent depression and could be an effective way of engaging adolescents in treatment. The evidence for family based treatments of adolescent depression is not well developed. The objective of this clinical trial is to determine whether a family based intervention can reduce rates of unipolar depressive disorders in adolescents, improve family functioning and engage adolescents who are reluctant to access mental health services.

**Methods/Design:**

The Family Options study will determine whether a manualized family based intervention designed to target both individual and family based factors in adolescent depression (BEST MOOD) will be more effective in reducing unipolar depressive disorders than an active (standard practice) control condition consisting of a parenting group using supportive techniques (PAST). The study is a multicenter effectiveness randomized controlled trial. Both interventions are delivered in group format over eight weekly sessions, of two hours per session. We will recruit 160 adolescents (12 to 18 years old) and their families, randomized equally to each treatment condition. Participants will be assessed at baseline, eight weeks and 20 weeks. Assessment of eligibility and primary outcome will be conducted using the KID-SCID structured clinical interview via adolescent and parent self-report. Assessments of family mental health, functioning and therapeutic processes will also be conducted. Data will be analyzed using Multilevel Mixed Modeling accounting for time x treatment effects and random effects for group and family characteristics. This trial is currently recruiting. Challenges in design and implementation to-date are discussed. These include diagnosis and differential diagnosis of mental disorders in the context of adolescent development, non-compliance of adolescents with requirements of assessment, questionnaire completion and treatment attendance, breaking randomization, and measuring the complexity of change in the context of a family-based intervention.

**Trial registration:**

Australia and New Zealand Clinical Trials Registry Title: engaging youth with high prevalence mental health problems using family based interventions; number 12612000398808. Prospectively registered on 10 April 2012.

## Background

There is increasing community and government recognition of the magnitude and impact of adolescent depression in Australia and worldwide. The Australian National Survey of Mental Health and Wellbeing conducted in 2007 suggested that approximately one in four adolescents experienced a mental disorder in the 12 months prior to the survey [1]. However, less than one in four of those attended a professional service during the preceding six months [2]. The incidence of mental illness in young people is the highest of any age group. Seventy-five percent of adults suffering from disorders such as substance use, mood, and anxiety disorders had an onset age before 24 years [3]. During adolescence, it has been estimated that between four and eight percent of youth meet criteria for a major depressive disorder in American studies, with a 20% cumulative incidence rate for depression in community samples by the age of 18 [4,5]. One of the most significant concerns in adolescent mental health is the elevated risk for suicide in adolescents with depression. Suicide is the second leading cause of death of young Australians aged 15 to 24 years [6]. Adolescent suicide victims are approximately six and a half times more likely to have been struggling with alcohol or drug abuse, and 27 times more likely to have been suffering from a major depressive disorder at the time of their death; compared with demographically matched community control participants [7].

These multifaceted and complex mental health problems, occurring within a developmental context and interacting with family factors, means that the traditional mental health service delivery and intervention models have limited success. This is compounded by the findings that less than a quarter of youth who have diagnosable mental health issues actually receive services [1,2]. In families where conflict and detachment are prevalent, parents may have a limited capacity to engage youth in treatments [8], while young people themselves often have little motivation to attend services [2]. An increasing number of young people currently grow up experiencing alcohol or drug use and mental health problems, resulting in prolonged financial and material dependence on their parents [9].

### Treatments for adolescent depression

The evidence for treatments of adolescent depression is less well established than for adult populations [8]. A number of comprehensive reviews and meta-analyses show that there is limited evidence and the existing findings have not established the efficacy for either pharmacological or current psychological therapies for youth depression, anxiety and comorbid alcohol or drug abuse [10-12]. A number of psychological therapies have been supported for use with depression, anxiety and alcohol or drug abuse in adult studies, and have some limited support for use with adolescents. The most well researched individual therapy for depression and anxiety are cognitive behavioral therapies (CBT) which are well supported as a treatment for depression and anxiety in adults, but the evidence for the efficacy of these treatments with youth is much weaker [8,13-15]. The key point, however, is that individual treatment models presuppose the willingness of an adolescent to recognize problems, engage with services and complete treatments, which is very frequently not the case [16].

### Family as a target for intervention

Family-based interventions may prove to be effective for depressed adolescents, by: (i) enhancing youth engagement, (ii) targeting interactions between family members and resolving conflict, (iii) increasing support and family cohesion, (iv) reducing exposure to stressors within the family, and (v) maintaining adolescents within protective family environments for a longer period. There is evidence to suggest that depressed youth who are also in conflicted parent-child relationships are less responsive to individual treatments [17]. There is also substantive evidence from cohort studies that risk factors for depressive and anxiety symptoms are predicted by poor parent-child relationships, high family conflict, poor family attachments and detachment from family activities [18,19]. Given the evidence for family influences, researchers have repeatedly called for the development of prevention and early intervention programs that target family factors including assisting parents to create a warm and supportive family environment, appropriate parental monitoring and the use of authoritative parenting approaches across adolescence [20]. The other major advantage of family-based interventions is that they allow an avenue by which young people who are initially reluctant to acknowledge problems or attend services can be gradually engaged via other family members attending the service and showing a motivation for change.

Based on clinical experience and prior feedback from local services in an Australian context, if a concerned parent contacts a youth mental health service but the young person is unwilling to engage in treatment, the service is often unable to offer a program for the family. At most, a parent support session or support group may be offered to parents. In many cases, parents are informed that unless their adolescent is willing to attend, no service can be offered.

The current randomized controlled trial was designed to evaluate the capacity of family based interventions to: i) reduce the depressive symptoms of young people (12 to 18 years) and improve the mental health of their parents; ii) engage adolescents reluctant to use mental health services individually by working therapeutically with the whole family unit; and (iii) improve family functioning in the context of adolescent depression.

### The development of the BEST MOOD intervention

The intervention to be evaluated is known as the Behavior Exchange Systems Training (BEST MOOD) program and has been developed over three distinct stages. The original BEST intervention was initially developed as a multifamily group education program for parents, to be delivered by a mental health professional trained in the model [21,22]. The content of the initial BEST program focused on alcohol and drug use by adolescents. This version of the program was shown to reduce parental mental health symptoms and family stresses [23]. To increase efficacy for the youth themselves, the second stage of development (BEST Plus) included siblings who join their parents in the group for the final four weeks of the eight week program. Evaluations showed additional positive changes in the family system were produced in mental health and stress symptoms, family cohesion and increases in action by young people to address their substance use [9,24-26]. The third major development of the intervention model, the current BEST MOOD intervention, involved further developing the program as a broader intervention suitable for adolescents who present with depressive disorders [27]. The development of BEST MOOD was motivated by the predominance of adolescent depression in community referrals to previous evaluations and our discovery of an increased motivation of depressed adolescents to attend the final four sessions of BEST Plus. Accordingly, content was designed specifically to invite the adolescent to attend parallel sessions with their parents for the final four sessions and for depressed adolescents to benefit therapeutically from these sessions. The additional content in BEST MOOD was designed to complement the messages delivered to parents concerning improved family functioning, stress regulation, improved communication and resolution of major life events in the family’s history. This paper describes the design of an RCT to evaluate BEST MOOD.

## Methods/Design

### Overview

The study is a multi-center, double-blind, randomized controlled trial comparing two group interventions: the BEST MOOD program and a treatment-as-usual supportive parenting program known as Parenting Adolescents Support Training (PAST). Both interventions are for families of youth who present with a unipolar mood disorder, here defined as major or minor adolescent depression or dysthymia. In both treatment conditions, families receive eight sessions of treatment delivered over two hours per week. The trial will run across several sites in both metropolitan Melbourne and the regional Victorian city of Geelong. Families will be recruited primarily from the intake service of a large government run mental health service in the eastern region of Melbourne (Eastern Health’s Child and Youth Mental Health Service; CYMHS), but community referrals will also be accepted from schools and community based health and mental health services, and via promotion of the study at community forums. At the completion of the interventions, face-to-face interviews will be conducted to better understand the level of engagement of participants with the treatment following intake/assessment. The aims of the qualitative component of the study are to explore the phenomena of not taking-up treatment, discontinuing treatment after commencement, completing treatment and benefiting from treatment, and the perceived influencing factors on these outcomes.

The primary outcome measure will be rates of remission of depressive disorder (major, minor, or dysthymia), according to a structured diagnostic clinical assessment, utilizing modules from the Structured Clinical Interview for *DSM-IV Childhood Diagnoses* KID-SCID; [28]. The secondary outcome measures will include: adolescent mental health (parent and self-reported), parent mental health, the young person’s level of engagement with mental health services, the parent-child relationship, and the therapist-client working alliance. Psychologists, social workers, and postgraduate psychology students who will be trained and supervised in one of the interventions for an equivalent amount of time, will deliver treatment. Therapists and assessors are blinded to the content of the alternate intervention, and are blinded to whether they are delivering the experimental or control condition in the study.

### Aims and hypotheses

The primary aim of the trial is to evaluate the efficacy of the two interventions as: (a) treatments for youth mental health problems and (b) as methods of engaging youth in treatments. The secondary aims will include: (c) to build capacity and increase service integration across several service delivery sectors by providing evidence based intervention programs for youth and their families; (d) to influence national and state government policy on the use of family based approaches in the field of youth mental health; and (e) make use of the study partnerships for national promotion and dissemination of family-based treatment models for youth mental health.

The primary study hypothesis is that youth in the BEST MOOD intervention will demonstrate significantly greater rates of remission of depressive disorders (major, minor, or dysthymia), than youth in the PAST intervention. Secondary hypotheses include: (a) that parental and youth mental health and relationships will improve in response to both interventions, but significantly more so in response to BEST MOOD; (b) that greater treatment engagement for youth will be associated with greater improvements in youth mental health outcomes; and (c) that youth in the BEST MOOD condition will report greater treatment engagement than youth in the PAST condition.

### Eligibility criteria

#### Inclusion criteria

Families will be included in the study where there is an adolescent aged 12 to 18 years who is currently presenting with a depressive disorder. Specifically, the young person must, at the time of assessment, meet the criteria for either major depressive disorder, minor depressive disorder, or dysthymic disorder as assessed via current *DSM-IV* criteria.

#### Exclusion criteria

Exclusion criteria for the study are listed in Table 1. Specifically, in terms of diagnostic exclusions, youth who currently report any of the following will be excluded: mania, hypomania, a bipolar disorder, psychosis or psychotic disorders, an intellectual disability, a pervasive developmental disorders, drug dependence other than alcohol, nicotine or cannabis use, or any severe mental illness currently requiring inpatient treatment. Other than these listed diagnostic exclusions, other forms of co-morbidity will be accepted into the study, as long as a unipolar depressive disorder is the primary presenting issue (where this is in doubt clinical assessment with the senior clinical psychologist, CI Andrew Lewis will determine eligibility). Other exclusions are if the parent/s or caregiver/s is/are unwilling or unable to attend and participate in a group, or if participating family members are unwilling or unable to complete the assessment process (as per Table 1).

**Table 1:** Exclusion criteria

**Individual exclusions**	**Family exclusions**
^a^Mania, hypomania, or bipolar disorder	A current child protection investigation
An intellectual disability or a severe mental illness requiring inpatient treatment or otherwise impairing their ability to participate in a group program	The young person is unwilling to undertake the minimum requirements for entry to the study including completion of the consent form, telephone KID-SCID interview, and the baseline questionnaire
An inability to understand spoken English	The young person is currently pregnant
The parents indicate they are unable to participate fully in the program, including attendance at group sessions one evening per week for eight weeks and completing all questionnaires	Insufficient address for follow-up or unwillingness to be followed-up
^a^Psychosis or psychotic disorders, including drug induced psychosis	The young person is currently fully engaged and regularly attending a treatment service for their mental health problems which the family considers are adequately addressing their mental health needs
Pervasive developmental disorder, including autism but not including Asperger’s syndrome	
^a^Drug dependence, with the exception of alcohol, nicotine or cannabis	

Notes: ^a^ A history of these criteria does not exclude parents/caregivers from the study provided they have been asymptomatic/stable for several months.

### Recruitment

**Figure 1:**
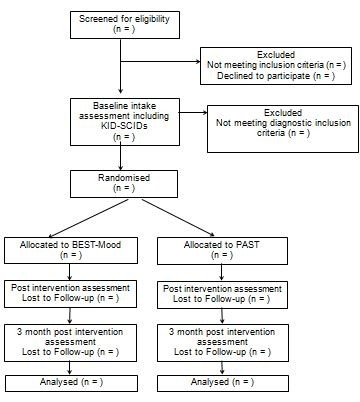
CONSORT flow diagram of progress through the phases of the Family Options trial.

The flow of participants from recruitment through to end of study is shown in Figure 1, as adapted from CONSORT guidelines. A multifaceted recruitment strategy will be employed to recruit participants. Intake workers will be co-located with a local youth mental health service (the Eastern Health Child and Youth Mental Health Service (CYMHS) Access Team). Schools and agencies will be provided with information on the research study (marketed as the *Family Options* (FO) program), educated on the signs and symptoms of mood disorders, and will be asked to refer relevant families to the program. The research team will also directly market to the public via media releases, development of a website, utilizing the existing promotion media of partnership agencies and group Emails to local service providers. The research team will also host free education seminars for parents on parenting challenging teens, as a recruitment strategy. This strategy was found to be an effective recruitment tool in the pilot study [26].

Enrollment into the trial will involve an intake worker conducting an initial telephone assessment with a parent or caregiver of the young person. This assessment will include: family demographic information, a genogram, a screen for inclusion and exclusion criteria, a the KID-SCID Mood Episodes Module B as reported by parents, and information on the young person and parent/s current mental health status and support services psychotropic medications, recent alcohol and other drug use and violence within the family, and risk assessments including suicide and self-harm risk. If the family is deemed eligible for participation in the trial, the intake worker will then confirm eligibility by administering selected modules of the KID-SCID to the young person. The selected modules are those for Major Depressive Disorder, Minor Depressive Disorder, and Dysthymic Disorder [28]. If the young person meets the inclusion criteria, the intake worker will then allocate to treatment condition using the randomized allocation sequence. Participants will be advised verbally and in writing that they will be free to withdraw at any stage upon request. Attempts will be made to assist families to find an alternative source of treatment, where possible, should they choose to withdraw at any stage.

### Randomization procedures and methods

Block randomization will be used following the procedure described in Altman and Bland [29]. It is desirable to have the number of participants allocated to each treatment condition similar at all times, in order to fill the treatment groups at approximately equal rates. The random number sequence will be generated by CI Lewis, in the following manner. Using blocks of four, there are only six possible ways of combining treatments A and B. Therefore for each sequence of four eligible families, both treatment conditions will be allocated twice. Six blocks will be generated. Using the website http://www.random.org, a random number series will be generated using numbers in the range of 1 to 6. A number will be allocated to each block. A second random number sequence will be generated for the allocation of blocks again. Blocks will then be arranged by going down each column until 200 conditions are allocated for Melbourne referrals, and a further 60 allocated for Geelong referrals. Finally a coin will be tossed where PAST = A and BEST-MOOD = B for heads, or PAST = B and BEST-MOOD = A for tails.

Sequentially numbered, opaque, sealed envelopes will be used to store the allocations, kept with the trial manager. Those allocating to treatment condition (intake workers) will be blinded to the randomization sequence and the overall study hypotheses.

### Planned interventions

#### Behavior exchange systems training - mood (BEST MOOD)

BEST MOOD is a fully manualized treatment that has been developed based on family-systems theory. It consists of an eight-week, professionally-led group program designed to assist concerned parents to effectively initiate family changes to reduce youth depression. The active components of the BEST MOOD program include: mental health literacy; clarification of roles within the family; clarification of family goals to reinforce parental vision and leadership; skills in family communication, positive reinforcement and boundary setting; encouraging parent/guardian self-care; stress reduction techniques, encouragement of family connectedness; and family homework tasks related to these treatment components. The parent/s initially receive four, two-hour sessions of weekly intervention, after which the adolescent (12 to 18) and siblings (aged 12 years of age and over) are invited to also attend with their parents and complete four additional, two-hour sessions of a multi-family weekly intervention. During these last four sessions there are whole group activities, and also activities in smaller groups of parents and adolescents separately.

### Parenting Adolescents Support Training (PAST)

The PAST intervention will act as the treatment-as-usual control condition in this trial. Parents will attend a professionally facilitated parenting group which uses supportive counselling techniques. The PAST intervention runs for eight, weekly two-hour sessions. Youth and siblings (aged 12 and over) are invited to join in the fifth week. This intervention has been manualized for the purpose of this study. The content of PAST has been designed to equate with standard practices in currently available services in Australia, where if any service is offered, it is likely to consist of a parent support group. The development of this intervention was guided by the current protocols for the family mental health support services in Victoria, Australia. The PAST intervention offers: (a) supportive counselling to assist parents to articulate and identify concerns and (b) psycho-education to increase parents’ knowledge about youth mental health problems. The main content of the PAST group is support and the opportunity to share experiences and ideas as well as receiving contact with a mental health professional.

### Assessments and outcome measures

The following measures will be administered to assess the previously described study hypotheses across three time points during the trial: enrollment in the trial (T1), completion of treatment (T2), and three months post-treatment completion (T3). To maximize the clinical validity of the outcome evaluations, these assessments will involve both clinician administered and self-report measures. These measures have been chosen based on piloting in previous trials and in terms of minimizing participant burden while maximizing sensitivity to clinical change across key domains.

### Screening measures

#### Demographics

Demographic and clinical information will be collected from parent(s)/caregiver(s) and youth, including presenting problems, mental health history and treatment, family structure, and current psychotropic medications.

#### Youth depressive disorders

The Structured Clinical Interview for *DSM-IV Childhood Diagnoses* (KID-SCID) [28] is a semi-structured instrument designed to generate childhood *Diagnostic and Statistical Manual of Mental Disorders Fourth Edition DSM-IV*[30] diagnoses for clinical research studies. This instrument is modeled on the extensively used adult version (SCID). The modular nature of the KID-SCID permits users to select modules relevant to their research. For the purpose of this trial, modules were selected to enable the differential diagnosis of major depressive disorder, minor depressive disorder and dysthymic disorder. The selected modules were then tailored for use, such that two versions were created: one for completion with the young person, and a brief screening version for completion with a parent/caregiver. The KID-SCID modules will be conducted via telephone with young people, to determine eligibility and to assess the primary study hypothesis.

Modules of the KID-SCID selected for use with young people in this study as follows: Current Major Depressive Episode B1; Current Manic Episode B6; Hypomanic Episode B10; Dysthymia B14; Mood Disorder Due to a General Medical Condition B18; Substance Induced Mood Disorder B20. Modules B6, B10 and B18 and B20 were included as the differential diagnosis of major depressive, minor depressive and dysthymic disorders requires information elicited in the completion of these modules. On the recommendation of KID-SCID developer Frederick Matzner, MD, the research team also used the following modules to the KID-SCID in the current trial: Past Major Depressive Episode, Past Manic Episode, Past Hypomanic Episode, and Major Depressive Disorder E4. These additional modules enable Current Mood Disorder Differential Diagnosis Module E to be completed. Mania, Hypomania and Bipolar Episodes and Disorders were only assessed to the extent that they could be ruled out for the purpose of accurate differential diagnosis of the depressive disorders.

Modules from the KID-SCID were also selected to form a proxy measure of major and minor depressive episodes or dysthymia for adolescents based on parent report. This will be conducted with the parent with regard to their adolescent. Included are: Current Major or Minor Depressive Episode; Dysthymia - single item; Mood Disorder Due to a General Medical Condition B18; Substance Induced Mood Disorder B20. The convergence of diagnosis via this parent proxy version of the KID-SCID and the adolescent administered KID-SCID will be assessed as part of the trial.

#### Youth psychotic symptoms

For differential diagnosis, psychosis will be screened using selected items of the Psychosis-Like Symptoms Interview PLIKS; [24]. Positive screens will be further assessed by a senior clinical psychologist to determine if the participant should be excluded on the basis of meeting diagnostic criteria.

### Parent, youth and sibling questionnaires

Participating families will also complete a set of questionnaires at baseline, post-group and at three month follow-up, in order to assess the secondary study hypotheses. Group attending parents/caregivers, the young person with depression symptoms, and their siblings aged 12 and over residing in the family home will all be invited to complete a set of questionnaires, as follows.

#### Youth and sibling mental health

*The Strengths and Difficulties Questionnaire (SDQ)* consists of 25 items [31]. The questionnaire will be reported by the parent about the youth with depressive symptoms, and the youth and siblings about themselves. The items load onto five subscales relating to youth mental health, including emotional symptoms, conduct problems, hyperactivity/inattention, peer relationship problems and prosocial behavior. *The Short Mood and Feelings Questionnaire (SMFQ)* consists of 13 items, which are administered to identify depressive symptoms amongst youth. The SMFQ is based on the 34-item version. Higher scores reflect more depressive symptoms [32]. The questionnaire will be administered to the parents (reporting about youth), youth (self-report), and siblings (self-report). A considerable amount of research indicates that the SMFQ and SDQ have good reliability and validity [33-35]. *The Alcohol Use Disorders Identification Test (AUDIT)* will be administered to identify recent problem alcohol drinking behavior [36]. The AUDIT has shown sound psychometric properties in prior research [37-39]. *The Depressive Experiences Questionnaire for Adolescents (DEQ-A shortened)* is a 20-item questionnaire is designed to assess youth depressive symptoms, and was developed from the 66-item DEQ for adults [40]. Items were identified from the DEQ according to their loading onto the three subscales; self-criticism, dependency and efficacy [41]. Adequate internal consistency has also been demonstrated (DEQ-A shortened self-criticism subscale alpha = 0.65, DEQ-A shortened dependency subscale alpha = 0.70, DEQ-A shortened efficacy subscale alpha not reported) [41].

#### Parent mental health

*The Depression Anxiety Stress Scales-21 (DASS-21)* is a 21-item measure which reflects three subscales including depressive symptoms (7 items), anxiety symptoms (7 items) and stress/tension items (7 items) experienced over the previous week (9), and will be self-reported by parents. The items from the original 42-item measure, which loaded most highly onto the subscales, were selected and reflect those included in the DASS-21 [42]. Comparable convergent validity has been found with other scales designed to measure depression, anxiety and stress or tension (that is, Beck Anxiety Inventory (BAI) and DASS-21 Anxiety subscale *r* = 0.85, Beck Depression Inventory and DASS-21 Depression subscale *r* = 0.79, BAI and DASS-21 stress/tension subscale *r* = 0.70) [43]. Good internal reliability has also been shown [43].

#### Familial relationships

*The Kansas Family Life Satisfaction Scales - modified (KFLS-M)* is a four-item questionnaire based on the original KFLS administered to parents, designed to assess the parent-perceived level of satisfaction in the relationships with the other family members [26]. *The Experiences in Close Relationships - short form (ECR-S)* is a 12-item questionnaire designed to measure attachment styles in adults, particularly avoidant and anxious attachment styles. The measure is based upon the 36-item Experiences in Close Relationships. In the current study, it will be used to measure attachment styles of both parents. *The BEST Parenting Questionnaire* is a purpose designed, 15-item questionnaire designed to assess parenting experiences, comprising of two subscales that signify emotional dependence on adolescent behavior and assertive parenting. The items were developed to assess changes in behaviors emphasized within the BEST program curriculum [22].

#### Youth engagement

A 19-item *Engagement Questionnaire* devised by the research team will be utilized to determine the youth’s use of mental health services, medication use, and potential barriers to service use. The items regarding barriers to and for service use items are responded to on a 7-point scale where 1 reflects ‘not at all important’ and 7 reflects ‘a very important factor’. *The University of Rhode Island Change Assessment Scale Readiness to Change Questionnaire- DELTA Project (URICA-D)* is a 12-item scale [44], and will be administered to youth with items adapted to reflect youth readiness to change in relation to their depression instead of in relation to alcohol and other drug problems. In addition, youth engagement will be assessed via their attendance or not at the group with their family when invited.

### Reimbursement

The questionnaire will be completed by the youth experiencing depressive symptoms, and with parental consent, their siblings (aged 12 and over) who reside in the family home. At each of the three time points, youth completion of a KID-SCID telephone interview and a full questionnaire will be reimbursed with AU$15 vouchers, while the siblings will be reimbursed with AU$10 vouchers for each questionnaire completed.

### Sample size and power

The trial aims to recruit a total of *n* = 160 participants. Based on pilot work and previously conducted clinical trials of family-based interventions compared to treatment-as-usual conditions, it has been estimated that it is possible to generate an effect size of *d* = 0.63 in reduction of mental health symptoms, which is equivalent to 14% increase in the odds of remission [45]. Out pilot data also indicates that treatment type by time interaction is able to produce up to 50% remission in depression diagnosis, over and above treatment type. With an alpha of 0.05 and power level of 0.90 for two-tailed tests, intraclass correlation coefficient (ICC) of 0.17 for within therapy group variation (estimated from our pilot data), and estimated 10% of variation in the main outcome accounted for by other variables in the model (treatment arm, time, parental socioeconomic status), the minimum sample size required equates to *n* = 57 per treatment arm. Based on pilot studies, an attrition rate of 20 to 25% is anticipated. Therefore, a minimum of 80 participants into each of the two treatment conditions will be recruited. Comparisons will be undertaken between those who drop out and those who are retained in order to assess for attrition bias.

With respect to our secondary hypotheses, anticipated final sample size of 114 individuals, will also allow us to detect small effect size (d = 0.23) for the treatment arm by time interaction for continuous outcomes, with 90% power and alpha level of 0.05, assuming ICC of 0.20 for within-person (between-assessment) and ICC of 0.07 for within therapy group variation (estimated from our pilot data).

### Statistical analysis plan

Repeated measures binomial data will be analyzed over three time points using a binary logistic regression within the Generalized Linear Mixed Model (GLMM). The model will be fitted in STATA (xtmelogit). For any continuous-scale data we will use a linear mixed model approach in STATA. For both types of data we will explore variance-covariance structures for the evaluations within an individual. Clinical significance and an index of reliable change will be calculated for each measure using the criteria of Jacobson and Truax [46]. Findings will be analyzed in terms of standardized mean differences and effect sizes achieved will be compared to existing high quality trials to determine relative efficacy of this intervention. Multivariable multilevel models will also explore other covariates on the relationship between treatment arm and study outcomes, with a view to improving the intervention and specifying its ideal target population. Data will be stored in a locked cabinet, and a password protected computer file will contain de-identified (coded) data. A proportion of the data will be double-entered, and range checks will be performed for accuracy checking. Intention-to-treat analysis will be used with the last observation carried forward as a conservative estimate of outcome when data are missing at follow-up.

In addition, analysis of qualitative data from the transcribed interviews of participants who did not take up the option of treatment following intake/assessment, discontinued treatment, or completed treatment will be undertaken. Thematic analysis of participants’ accounts of their experience of the intake/assessment process and reasons for either engaging with or distancing themselves from the treatment offered will be undertaken. Meanings participants assign to the process will also be extracted from the data. Analysis of the data relevant to the phenomena of interest will assist in the identification of barriers to engagement and change for families.

### Ethics

The study protocol has been approved by Eastern Health Human Research Ethics Committee, Victoria, Australia; and was then approved by Deakin University Human Research Ethics Committee, Victoria, Australia. All participants sign written consent forms, and are given verbal and written full informed consent statements, approved by the ethics committee (see Additional file 1). Details of any possible risks, benefits and reporting of harms are included in this explanation, and this occurs at the initial point of contact with participants (prior to commencing the assessment). No harms are expected from participation in this trial. Participants are given contact details for an external person on the ethics committee for complaints, independent from investigators and the sponsor. Protocol modifications will be communicated to relevant parties (for example, investigators, trial participants, journals, ethics committee) in written form.

## Discussion

There are many challenges in conducting clinical trials in adolescent mental health. These include diagnosis and differential diagnosis of mental disorders in the context of adolescent development, non-compliance of adolescents with requirements of assessment, questionnaire completion and treatment attendance, breaking randomization, and measuring the complexity of change in the context of a family-based intervention.

To assess the trial outcomes in the most convincing manner the research team opted to diagnose depression in adolescents using a structured interview to ensure that diagnostic criteria are met. This increases demand on participants and requires extensive training of the assessment team, as well as monitoring of the quality of such assessments. In the context of adolescent depression, the use of structured clinical assessments can be challenging since this is perceived as an onerous task by adolescents who often are not forthcoming about their mental health symptoms. Operationalizing outcomes on the KID-SCID as the primary outcome of the clinical trial is therefore not without risk; these include challenges of high attrition and missing data as well as the challenge of recruiting adolescents willing to undertake such assessments. To mitigate these risks, we developed a parent proxy version of the KID-SCID which will be administered to a primary caregiver, and we plan to assess its accuracy compared with the youth report version.

Another notable feature of our early experiences in setting up this trial has been the relatively high rate of initial reports of psychotic-like experiences within a cohort of depressed adolescents completing the KID-SCID. Upon further clinical assessment these experiences often do not meet full criteria for psychotic disorders, and are often fleeting experiences or experiences which are phenomenologically distinct from psychotic symptoms.

In terms of inclusion and exclusion criteria, the decision was made to exclude certain mental disorders that were likely to make participation difficult or render treatment less effective, for either parents or youth. As described above, exclusions are generally limited to severe mental disorders and drug dependence to major forms of substance abuse which would impede participation in the groups. Therefore we will not exclude youth or parents who have used alcohol, nicotine or cannabis. For parents, we accept parents with both common and severe mental disorders so long as clinical assessment indicated that the condition is well managed and does not require inpatient treatment.

Co-morbidity is common in adolescent mental health. In this study we elected to accept youth with mental disorders with depression on the condition that a depressive disorder is the primary presenting issue. This presents a clinical challenge in many cases which can be unraveled by careful clinical assessment of the course and duration of the various presenting conditions and considering information provided by the parent or other professionals. Problems such as eating disorders, substance misuse and conduct or behavioral problems are likely to be co-morbid and have not necessarily been excluded in the current trial.

Collecting data from adolescents is a well recognized challenge and the participant demands within a RCT are considerable. Added to this challenge is the high rate of family conflict, characteristic of adolescent mental health conditions, operating as a bidirectional influence that is both a contributing cause and consequence of adolescent mood disorders. We have encountered many circumstances in a previous trial [24,25] in which parents are unable to convince adolescents even to provide consent or undertaken a minimal amount of assessment in order to participate in the trial. The current trial has sought to address this issue with the use of strategic incentives, a staged assessment process and the use of parent proxy reports on adolescents wherever possible.

In terms of study design, we have been mindful of the challenges inherent in randomizing to treatments that use different modalities. One of the challenges in the field of adolescent mental health mentioned in the introduction is that the bulk of the existing evidence is based on individual adolescent psychological therapies, primarily CBT. In our previous attempt to conduct a head-to-head comparative trial of CBT versus BEST Plus, we found that parents randomized to the CBT condition whose adolescents refused to attend were strongly inclined to break the randomization and seek attendance to the BEST Plus condition. To address that issue, in this trial we offered equivalent group interventions for parents. While this has improved the problems of breaking randomization, it has also resulted in challenges in terms of obtaining adequate numbers to commence groups as scheduled.

Finally, the current trial of a family based intervention is based on a systemic model of therapeutic change. The measurement of outcomes needs to consider changes both in parents and in adolescents, and in terms of overall family dynamics. This is a more complex set of outcomes than one would find in a trial of an individual psychological therapy and is reflected in the set of measures of both outcomes and processes chosen in the trial.

## Trial status

The trial is currently underway with participants currently being recruited and assessed. Date of first enrolment is 17 October, 2012. Estimated completion date is late 2014.

## Abbreviations

AUDIT: Alcohol Use Disorders Identification Test; BAI: Beck Anxiety Inventory; BEST: Behavior Exchange Systems Training; CBT: cognitive behavioral therapy; CI: Chief investigator; DASS-21: Depression Anxiety Stress Scales-21; DEQ-A: Depressive Experiences Questionnaire for Adolescents; ECR-S: Experiences in Close Relationships-short form; FO: Family options; GLMM: Generalized Linear Mixed Model; ICC: Intraclass correlation coefficient; KFLS-M: Kansas Family Life Satisfaction Scales - modified; KID-SCID: Child and adolescent version of the structured clinical interview for the *DSM-IV*; PAST: Parenting Adolescents Support Training; PLIKS: Psychosis-like Symptoms interview; RCT: Randomized controlled trialSDQ, Strengths and Difficulties Questionnaire; SMFQ: Short Mood and Feelings Questionnaire; URICA-D: University of Rhode Island Change Assessment Scale Readiness to Change Questionnaire-DELTA project.

## Competing interests

The authors declare that they have no competing interests. The authors hold intellectual property responsibility for the interventions under study.

## Authors’ contributions

AL - CI, senior trial manager, senior clinical psychologist, supervisor, co-developer of interventions. MB - trial manager, clinical psychologist, supervisor, co-developer of interventions. JS - intake manager and worker, blinded to interventions (and to sections of this paper during writing and publication). LS - research assistant, no other roles in the study. NB - data manager, facilitator of one group, blinded to content of the other group (and to sections of this paper during writing and publication). DL - CI, advising on management group on intervention development, design, analysis and reporting and contributed to editing this paper. TK - CI, advising management group on intervention development, design, analysis and reporting and contributed to editing this paper. JWT - CI, advising management group on intervention development (co-developed BEST Plus), design, analysis and reporting and contributed to editing this paper. All authors read and approved the final manuscript.

## Supplementary Material

Additional file 1 Participant Information and Consent Forms. Click here for file

## Access to data and publications

All CIs will have equal access to, and will collectively decide upon, who shall have access to the completed dataset, and how the data shall be used. Results shall be submitted for publication in peer reviewed journals and conferences, prepared by the CI’s and research team, and potentially with the assistance of postgraduate students at Deakin University and paid research assistants.
