# Supplementary material for: Adolescent depressive disorders and family based interventions in the family options multicenter evaluation: study protocol for a randomized controlled trial
Source: Trials. 2013 Nov 13;14:384. doi: 10.1186/1745-6215-14-384 (PMC3842795; doi:10.1186/1745-6215-14-384)
Supplement: Additional file 1 — Participant Information and Consent Forms. [file 1745-6215-14-384-S1.doc]

**Additional file 1: Participant Information and Consent Forms**

**PARTICIPANT INFORMATION and consent form (PICF) For Parents/guardians**

Participant Information and Consent Form: For multiple Eastern Health sites, including Turning Point Alcohol and Other Drug Services and CYMHS; Deakin University; Headspace Collingwood; and for *drummond street services*

Date: January 2012

**Full Project Title:** Engaging youth with high prevalence mental health problems using family based interventions

**Brief Project Title:** Family Options Study

**Principal Researcher:** A/Prof Andrew Lewis

**Associate Researchers:** Prof John Toumbourou, Dr Tess Knight, Dr Melanie Bertino, Prof Dan Lubman, Dr Steven Leicester

**Student Researchers:** Julian Thompson, Ai Tran, Joanna Skewes, Milanda Matthews and Jaclyn Danaher

This Plain Language Statement and Consent Form is 8 pages long. Please make sure you have all the pages.

1. Introduction

You are invited to take part in this research project. This is because you have contacted us with concerns regarding your young person’s mental health and your interest in attending a counselling group about these issues. The research project aims to compare the mental health and engagement of young people after their family has participated in a counselling group.

This Participant Information and Consent Form tells you about the research project. It explains what is involved to help you decide if you want to take part.

Please read this information carefully. Ask questions about anything that you don’t understand or want to know more about. Before deciding whether or not to take part, you might want to talk about it with a relative, friend or your local health worker.

Participation in this research is voluntary. If you don’t wish to take part, you don’t have to.

If you decide you want to take part in the research project, you will be asked to sign the consent section. By signing it you are telling us that you:

• understand what you have read;

• consent to take part in the research project;

- consent to be involved in the procedures described;
- consent to the use of your personal and health information as described.

You will be given a copy of this Participant Information and Consent Form to keep.

2. What is the purpose of this research project?

This project aims to compare two counseling groups to determine if working with the family can help to reduce young people’s depression and other mental health symptoms. We also aim to evaluate the ability of the groups to engage young people into a treatment for mental health, and to improve parents’ and siblings’ mental health and wellbeing. This is important research because it will advance our understanding of what treatments work best for young people with these mental health issues. This is likely to lead to better care for families like yours in the future.

Research suggests that approximately 80 percent of young people with a mental illness do not receive any suitable treatments, and that many young people are reluctant to attend a treatment for their mental health problems. This can be challenging for families. A few studies also suggest that working with the families of youth with depression or drug and alcohol problems can have a positive impact on young people and other family members. However more studies are needed to confirm this finding. Also there is almost no past research about whether young people are more likely to attend a treatment after their parents have attended a counseling group.

Overall, we hope that 180 families will participate in this project over the next two years. Most of these (up to 145 families) will take part at one of the Eastern Health or Deakin sites, and the rest (approximately 35 families) will take part at *drummond street services* site. There are two different groups in the study. Each group is designed for parents of youth with mental health symptoms. Both groups provide support and education to parents. During both groups your young person will be invited to join in at a later stage. The project is only running in Melbourne and Geelong, Australia at the sites listed on this form. This research project builds on earlier research done by a team of people at Deakin University and *drummond street services* in collaboration with *beyondblue* – comparing a one on one youth treatment with a family group for youth depression. The group treatment was well received and lead to improvements in the mental health of young people and their families. Four of the researchers from this earlier project are the investigators for the current project. Two new researchers have also joined the team, Professor Dan Lubman and Dr Steven Leicester from Eastern Health. This research has been funded by: *beyondblue,* the Australian Drug Foundation, *drummond street services,* Deakin University and an Australian Research Council linkage grant.

The following people are student researchers who will be working on the project: Julian Thompson, Ai Tran and Jaclyn Danaher. These students are postgraduate students completing the professional masters or doctoral program in psychology at Deakin University. The students will assist the project by helping with assessments, groups, and data, and writing up parts of the research outcomes for their thesis which will count towards their academic record.

3. What does participation in this research project involve?

**Procedures**

If you chose to take part in this project the following steps will take place:

1. You will complete a brief intake interview over the phone to get an initial idea about your eligibility. During this phone call the study will be explained to you, and you will have the chance to ask any questions. This intake call usually takes about 30 minutes. If you wish to participate after that, you will be asked about your family details and demographics, and your primary concerns, and some other questions about the study criteria relating to mental health of family members. You will also be asked for permission to contact your young person to do a telephone screen for depression, preferably at a time when you are home together.
2. Your young person will be contacted. During this phone call the study will be explained to them, and they will have the chance to ask any questions. If they wish to participate after that, they will be asked to complete a phone screening measure of depression. This measure asks about symptoms of depression. For this reason, it is preferable if you are home with your young person when they complete this measure.
3. Entry to the study depends on the number of symptoms of depression that your young person experiences. We will inform both you and your young person of the outcome of this interview, by informing both of you if they show some of the symptoms of depression or not. This is not a diagnosis, but just a research screening measure.
4. If the researchers identify any risk issues for your young person or your family, such as risk of serious harm to self or others, they will work with the relevant members of your family to develop a safety plan under the supervision of the senior psychologist.
5. If your family is eligible based on the phone screening responses, you will be asked to complete a consent form, giving consent for both yourself and your child (aged 12-18) to participate. Your child will also be asked to complete their own consent form. You will be asked to complete three sets of questionnaires on three occasions over the next 8 months – one set now, one set after you finish attending the 8 week group, and again 6 months later. The questionnaires ask detailed and sensitive questions about your demographics (e.g. age, gender, family make up), your own and your child’s mental health, health, and your own and your young person’s drug and alcohol use, and your relationships. Each set of questionnaires should take you approximately 30 minutes to complete. Your child will also be asked to complete several written and telephone surveys, for which they will receive reimbursements in the form of vouchers (to a maximum total value of $45).

If your family is ineligible based on the phone screening responses, or if you wish to withdraw at any time, you will be provided with alternative referral options. The results of the phone surveys will be placed in your confidential file along with some brief notes on what occurred and will later be destroyed along with all of the research data after 15 years. More information about the storage of your data is provided below (see section 8 of this form).

1. For eligible families, once we have received your consent forms and questionnaires, you will be allocated to a group. If you would like assistance completing these surveys, the researchers can arrange for you to visit the university, or for us to visit your home to assist you. When the group is full you will be informed of the starting date and we will endeavor to set the times for the groups to suit all participating families. The groups will probably run on a weeknight in the early evening. The groups will run weekly for 8 weeks during the school terms, for 2 hours each time. You will be invited to attend the sessions, at an Eastern Health site, Deakin, or *drummond street services.* Given that participants must be randomly allocated to a treatment, there will be limited capacity for you to choose a venue. However if the site is a greatly inconvenient you may wait for another group closer to home if there will be one available. A member of the research team will discuss this with you.

This study uses an ‘active control’ condition, meaning that both groups are expected to produce a beneficial response, and we are interested if one group is of greater benefit than the other to participants, or if they are of equal benefit. Therefore you will be randomly allocated to a condition by the research team and will not be able to choose which group you will enter.

Your personal information that is provided to the research team will be kept in a secure location as described below. Should you wish to access your personal data the research team can assist you to do so. However your child’s information will be provided to them individually upon request but otherwise kept private, unless they provide express written consent to release the information to you or any third party, or as required by law or ethical obligation (e.g. the need to protect them from harm). In this case these same procedures for releasing the information can be undertaken. It is also important to understand that the group sessions will be audio-taped. This will allow the research team to evaluate how well the counselors have delivered the group content, according to the way that it was designed. A selection of the telephone interviews will also be recorded for quality assurance purposes, with your verbal consent prior to doing so. These tapes will only be listened to by members of the research team. Recordings will be kept as a single copy, in a secure locked format, and will not be shared with anyone external to the research team. These records will be destroyed after 15 years of secure storage. Appropriate systems within the University will ensure this.

**Reimbursement**

You will not be paid for your participation in this research, however the group sessions will be provided to you free of charge. Your child will receive reimbursements for either a) returning the form to indicate that they do not wish to take part (one $15 voucher), or b) returning their completed consent form and all surveys (one $15 voucher per phone + written survey completed and returned). The maximum reimbursed amount that one child can therefore receive is $45 worth of vouchers over the 8 month participation period.

In cases where families complete an intake assessment, but do not subsequently proceed to the 8 week intervention groups, we will offer parent/caregivers voucher reimbursements of $10 each for taking their time to complete the pre-group questionnaires. Collecting this questionnaire data from willing parents will provide important research information about those who entered the study versus those who did not.

4. What are the possible benefits?

Possible benefits include receiving free assessment and treatment with an evidence-based method that is likely to improve your mental health and decrease stress, and which may improve your son/daughter’s symptoms of depression, anxiety or substance use. However, we cannot guarantee or promise that you and your son/daughter will receive any benefits from this project. This study is also important for the provision of effective treatment in the future.

5. What are the possible risks?

It is important to note that the researchers cannot guarantee absolute confidentiality with respect to illegal behaviour of which they are made aware. When talking with the researchers, including during the group sessions (which are audio taped), you should talk in general terms only; do not give any factual details about illegal activities, to avoid incriminating yourself or your young person.

Other than this, it is not anticipated that you will experience any risk or side effects directly from being involved in this study as you will be in a supportive environment and receiving treatment. If you become upset or distressed as a result of your participation in the research, the researcher is able to arrange for alternative counselling or other appropriate support. Any counselling or support will be provided by staff who are not members of the research team.

Other helpful sources of independent counselling should you experience discomfort include your local doctor, DirectLine 1800 888 236, ParentLine 13 22 89, LifeLine 13 11 14, or [Kids Help Line (for your child) 1800 55 1800](http://www.kidshelp.com.au/). DirectLine provides 24-hour, 7-day counselling, information and referral. At DirectLine, you can talk to professional counsellors who are experienced in alcohol and drug-related matters. DirectLine is free, anonymous and confidential. Parentline provides a statewide telephone counselling service to parents and carers of children aged from birth to eighteen years. LifeLine also provides 24-hour, 7-day counselling, information and referral. It is also staffed by trained professional counsellors and offers mental health and crisis support. Kids Help Line is a free and confidential telephone counselling service for 5 to 25 year olds in Australia. Kids Help Line is Australia's only free, confidential and anonymous helpline available for children and young adults.

**6. Do I have to take part in this research project?**

Participation in any research project is voluntary. If you do not wish to take part, you do not have to. If you decide to take part and later change your mind, you are free to withdraw from the project at a later stage. If you decide to withdraw, please notify a member of the research team. This notice will allow that person or the research supervisor to inform you if there are any special requirements linked to withdrawing. If you decide to leave the project, the researchers would like to keep the personal and/or health information about you that has been collected. This is to help them make sure that the results of the research can be measured properly. If you do not want them to do this, you must tell them before you withdraw from the research project. Your decision whether to take part or not, or to take part and then withdraw, will not affect your relationship with the researchers or Eastern Health, Deakin University, *drummond street services*, or any other service involved in this research.

7. How will I be informed of the final results of this research project?

The results of this project will be published in several scientific journals. Please email [andrew.lewis@deakin.edu.au](mailto:andrew.lewis@deakin.edu.au) for a summary of the findings or copy of any report. The final results should be available by December 2013.

8. What will happen to information about me?

Any information obtained in connection with this research project that can identify you will remain confidential and will only be used for the purpose of this research project. The information we collect will be stored in a locked cabinet within the School of Psychology at Deakin University for a minimum of 15 years from the date of publication, after which time it will be securely destroyed.

Your information that is entered into the computer database, including the youth phone interview about depression symptoms and the questionnaires that you each complete, will be assigned a code to protect your privacy. This information will be ‘re-identifiable’ – that is, your non-identifiable information in the computer database will be assigned a code, and can be linked back to your identifiable information using this code. Therefore we can ‘re-identify’ this information as yours if the need arises. The code will also be used to match up any new information that you provide with your existing information.

Apart from the computer database, there will also be a ‘hard copy’ research file in a filing cabinet, containing: your consent forms, contact and personal details, questionnaires, and written answers to the phone interview with your young person. There will also be one copy of a list which matches your assigned code for your research file with your data in the computer database, and this list will also be stored in the locked cabinet at Deakin University.

The researchers cannot guarantee absolute confidentiality with respect to illegal behaviour of which they are made aware. A serious and imminent threat to yourself or others may be subject to reporting by a third person. Any information concerning protective safety of children is also subject to reporting to relevant authorities. Confidentiality of information you provide will be safeguarded except where the disclosure is required, authorised and permitted under law. In the event that your confidentiality needs to be breached, the researcher will discuss this with you prior to any action being taken. The researcher will also discuss this with the Principle Investigator prior to any action being taken.

Only members of the research team will have access to your files. In any publication and/or presentation, information will be provided in such a way that you cannot be identified, except with your permission.

9. Can I access research information kept about me?

## *In accordance with relevant Australian and/or Victorian privacy and other relevant laws, you have the right to access the information collected and stored by the researchers about you. Please contact one of the researchers named at the end of this document if you would like to access your information. In addition, in accordance with regulatory guidelines, the information collected in this research project will be kept for at least 15 years.*

10. Is this research project approved?

## *The ethical aspects of this research project have been approved by the Human Research Ethics Committees of Deakin University and Eastern Health. This project will be carried out according to the National Statement on Ethical Conduct in Human Research (2007) produced by the National Health and Medical Research Council of Australia. This statement has been developed to protect the interests of people who agree to participate in human research studies.*

11. Who can I contact?

The person you may need to contact will depend on the nature of your query. Therefore, please note the following:

**For further information or appointments:**

If you want any further information concerning this project or if you have any problems which may be related to your involvement in the project (for example, feelings of distress), you can contact the principal researcher

Name: Associate Professor Andrew Lewis

Position: Principle Researcher

Telephone: 92546774

Alternatively you can contact any of the following people who are associate researchers on this project: John Toumbourou 52278278, Tess Knight 92546595, or Melanie Bertino 92517364.

For after hours support you can contact your local doctor if available, or you can phone one of the telephone counselling services previously described (DirectLine 1800 888 236, LifeLine 13 11 14, or [Kids Help Line (for your child) 1800 55 1800](http://www.kidshelp.com.au/)).

**For complaints:**

If you have any complaints about any aspect of the project, the way it is being conducted or any questions about being a research participant in general, then you may contact:

Ethics Chairperson, Eastern Health Human Research and Ethics Committee, Phone 03 9895 3398, Email ethics@easternhealth.org.au

Consent Form: For Parents / Guardians

I have read, or have had this document read to me in a language that I understand, and I understand the purposes, procedures and risks of this research project as described within it*.*

I have had an opportunity to ask questions and I am satisfied with the answers I have received.

I freely agree to participate in this research project, as described.

I also freely agree for my adolescent/young adult (aged 12-18) to participate in this project according to the conditions in the Plain Language Statement.

I consent to the audio recording of treatment sessions, and the telephone interviews conducted with parent/s and adolescent family members involved in this study. I understand that these recordings will only be accessed by research team members, will not be copied or distributed, and will be kept in a secure locked location. I understand that the researchers have agreed not to reveal my own or my family’s identity and personal details without my consent, unless if required by law.

I understand that I will be given a signed copy of this document to keep.

Participant’s (your) name (printed) …………………………………………………………………………

Adolescent/Young Adults’ name (printed) ………………………………………………………………….

Your relationship to the above named Adolescent/Young Adult: ………………………………………..

Signature Date

Declaration by researcher*: I have given a full written explanation of the research project, its procedures and risks and I believe that the participant has understood that explanation.

Researcher’s name (printed) ………………………………………………………………………………..

Signature Date

*Note: All parties signing the consent section must date their own signature.*

PARTICIPANT INFORMATION and consent form (PICF) For ADOLESCENTS/YOUNG ADULTS (12-18)

Participant Information and Consent Form: For multiple Eastern Health sites, including Turning Point Alcohol and Other Drug Services and CYMHS; Deakin University; Headspace Collingwood; and for *drummond street services*

Date: January 2012

**Full Project Title:** Engaging youth with high prevalence mental health problems using family based interventions

**Brief Project Title:** Family Options Study

**Principal Researcher:** A/Prof Andrew Lewis

**Associate Researchers:** Prof John Toumbourou, Dr Tess Knight, Dr Melanie Bertino, Prof Dan Lubman, Dr Steven Leicester

**Student Researchers:**

Julian Thompson, Ai Tran, Joanna Skewes, Milanda Matthews and Jaclyn Danaher

This Plain Language Statement and Consent Form is 8 pages long. Please make sure you have all the pages.

1. Introduction

You are invited to take part in a research study. This study invites parents/families to come along to a group program with other families. **Parents come once a week for 2 hours, and young people will be invited to come to later sessions as well – but you do not have to attend with your parent if you do not want to.** You have been invited to take part in this research study because your parent/s want to try to make things better at home for you and your family by taking part in a counselling group with other parents. If you chose to participate in this research you will be invited to join in the group with your parents, but you do not have to if you don’t want to. This research study aims to compare how parents and young people are feeling both before and after their parents/family has participated in the counselling group.

This Participant Information and Consent Form tells you about the research project. It explains what is involved to help you decide if you want to take part in this research.

Please read this information carefully. Ask questions about anything that you don’t understand or want to know more about. Before deciding if you want to be in this study, you might want to talk about it with a family member, a friend, or your local health worker.

Participation in this research is voluntary. If you don’t wish to take part, you don’t have to.

If you decide you want to take part in the research project, you will be asked to sign the consent section. By signing it you are telling us that you:

• understand what you have read;

- agree to take part in this research study;
- agree to be involved in the research procedures including:
  - doing three brief written surveys and phone interviews with a researcher, before, after and six months after the group, and
  - you can also choose to attend the group with your parents at a later date if you want to.

By signing this consent form, you are also telling us that you:

- agree to the researchers collecting information from you and using it for research in the way that is described below.

You will be given a copy of this Participant Information and Consent Form to keep.

2. What is the purpose of this research project?

This research study aims to compare two counseling groups to find out if working with the parents/family in these counseling groups can help to reduce young people’s depression or other mental health problems. Another aim of the study is to find out if the groups can help young people to get connected with a mental health service should they want to. We are also looking at studying whether the counseling groups can help parents to have better mental health. This is important research because it will **help us to better understand what treatments work best for parents and young people** with these mental health concerns. This is likely to lead to better care for other families in the future.

Research suggests that approximately 80 percent of young people with a mental illness do not receive any suitable treatments, and that many young people are reluctant to attend a treatment for their mental health problems. A few studies have shown that working with the families of youth with depression or drug and alcohol problems can have a positive impact on young people and other family members. However more studies are needed about this. Also very few or no studies have been done about whether young people are more likely to attend a treatment for depression after their parents have attended a counseling group first.

Overall, we hope that 180 families will take part in this research study over the next two years. Most of these families (up to 145 families) will take part at one of the Eastern Health or Deakin sites, and the rest (about 35 families) will take part at the *drummond street services* site. There are two different groups in the study. Each group is designed for parents of youth aged 12-18. Both groups provide support and education to parents. During both groups young people will be invited to join in at a later stage. The project is only running in Melbourne and Geelong, Australia at the sites listed on this form.

This research study builds on earlier research done by a team of people at Deakin University and *drummond street services* in partnership with *beyondblue: the national depression initiative*. The earlier project compared a one-to-one youth treatment with a family group for youth depression. The group treatment was well received and helped young people and their families to feel better overall than before the group began. Four of the researchers from this earlier project are also working on this study. Two new researchers have also joined the team, named Professor Dan Lubman and Dr Steven Leicester from Eastern Health. This research has been funded by: *beyondblue,* the Australian Drug Foundation, *drummond street services,* Deakin University and the Australian Research Council.

The following people are student researchers who will be working on the project: Julian Thompson, Ai Tran and Jaclyn Danaher. These students are postgraduate students completing the professional masters or doctoral program in psychology at Deakin University. The students will assist the project by helping with assessments, groups, and data, and writing up parts of the research outcomes for their thesis which will count towards their academic record.

3. What does participation in this research project involve?

**Procedures**

If you choose to take part in this project the following steps will happen:

1. Your parent or guardian will tell us some information about you and your family over the phone. Your parent or guardian will give permission to contact you to do a short telephone interview with you for depression symptoms, at a time when you are home together.
2. We will call you. We will talk about the study to you. You can ask us any questions that you have. If you still want to participate after that, we will ask you the interview questions about symptoms of depression. Please tell us and/or your parents if you are upset or worried at any time, or if you want to stop.
3. If the researchers are very worried about the safety of you or your family because of what you or your parents tell us, they will work with services and/or the members of your family to develop a safety plan to try to keep everyone safe.
4. After you answer the questions about depression symptoms, we will then tell you and your parent whether you had enough depression symptoms to join the group. This does not mean you have depression; it is just about how many signs you have, for the research project.
5. If you do not have enough signs for this research project, we will try to help your family to find something else to help.
6. If you do have enough signs of depression, then we will send you a consent form (included with this form) and some questionnaires. We will ask you to fill in the last page of this form, and sign it, and also to fill in the questionnaires, and then return it all to us by post. You may also choose to do these forms online where the option is available. Your parent/s and siblings aged 12+ will also be asked to complete their own consent form and questionnaires. Then, your family will be invited to go into a group in this research project.

If you would like assistance completing these surveys, the researchers can arrange for you to visit the university, or for us to visit your home to assist you.

1. We will invite you to complete another phone survey about depression, and another written survey, two more times – at the end of the group and six months after the group.

The written surveys ask about your general and mental health (such as your moods and feelings, your activities, and your sleeping patterns, and recent drug use). Each survey booklet should take about 25 minutes for you to complete. **You will receive a $15 Coles-Myer, JB Hi-Fi or iTunes voucher for each completed booklet**. The information from your phone interview and your written surveys will be securely stored in your file.

Once you have completed the surveys and we have received your completed consent form, your parent/s will also need to return their surveys and then they will be put into a group. When the group is full your parents will be told the time and date when it will start. The groups will probably run on a weeknight in the early evening. The groups will run weekly for 8 weeks during the school terms, for 2 hours each time. You will be invited to attend the group with your parent/s after about 4 weeks if you want to, but you do not have to if you don’t want to*.*

If you decide not to do any of these things listed above, it will not in any way affect your relationship or your parents’ relationship with any of the people or agencies that are running this study.

**Groups**

There are two different groups running as part of this study, and your parents will be put into one of the groups if you decide to take part. We think both groups will be helpful for parents and families. We are studying them to find out if one group is more helpful than the other, or if they are the same. Families will not get to choose which group they go to, this will be allocated by the researchers in a random order. Putting people in to groups in a random order helps the researchers to make sure that the final results of the study are more accurate and not influenced or changed in unscientific ways by the people doing the research.

**Personal information**

- Your personal information that you give to the research team will be kept in a secure and locked file (including your personal details, consent form, and your survey responses). If you want to access your personal information, the research team can help you to do that. Your information will be kept private unless you want the researchers to give your private information to another person, including your parent/s. If you do want the research team to give another person your information, you must ask for this in a letter or email and send it to the researchers. Sometimes in rare cases, some of your private information may also be released without your approval, but only if it was required by law or of there was an ethical obligation (e.g. to protect you or other people from coming to serious harm) – see section 8 of this form for more information.

It is also important to understand that the group sessions will be audio-taped. This will allow the research team to see how well the counselors have run the groups as they are supposed to be run. A selection of the telephone interviews will also be recorded for quality assurance purposes, with your verbal consent prior to doing so. These recordings will only be listened to by members of the research team. Recordings will be kept as a single copy, in a secure place, and will not be shared with anyone outside of the research team. If you participate in this study, your written and phone survey responses and your consent form with your name on it will be stored in a separate, locked file at Deakin University. All of these things will be safely destroyed after 15 years.

**Reimbursement**

The group sessions will be provided to your family free of charge. You will not be paid for your participation in this research, but you will be reimbursed for your time spent on this research. You will receive $15 Coles-Myer, iTunes or JB-HiFi vouchers in return for your time.

a) If you return the consent form with only the bottom section completed, telling us that you do not wish to take part at all in the study 🡪 you will receive one x $15 voucher, and your family will not participate in the study,

b) If you return your completed consent form and also complete the phone and written surveys 🡪 you will receive a $15 voucher for each set of returned surveys, and your parents/family may choose to attend a group program

So, the maximum amount of vouchers that you can receive will be 45 dollars worth - for doing all of the phone and written surveys over the 8 months of the study. Your sibling/s aged 12 and over may also choose to complete the same surveys as you, and thy will also receive 15 dollars per returned survey. Your sibling/s aged 12 and over can also attend the group with you and your parents, if you want them to come.

4. What are the possible benefits?

Possible benefits of being in this research include receiving free mental health services (including assessment and treatment), including a program that is likely to improve your parent’s mental health and reduce stress, and which may also improve your mental health. However, we cannot guarantee or promise that you and your parent/s will receive any benefits from this project. This study is also important for knowing how to best help other families in the future.

5. What are the possible risks?

If you tell the researchers the details or facts about an illegal activity, they cannot promise to keep this secret from the authorities. So you should not give any specific details about crimes or illegal activities at any time to the researchers, including during the groups (e.g. who was there, what happened, where it happened). If you do, this could be used against you or your family.

We do not expect that there will be any other risk to you or side effects from being part of this research. Your family will be in a supportive environment and receiving a treatment group. If you become upset or bothered because of the research, the researcher can help you to find other counselling or other support if you want it. Any other counselling or support can be provided by different staff who are not members of the research team.

As well, if you feel upset these are some other helpful sources of outside counselling: your local doctor, [Kids Help Line (Phone 1800 55 1800](http://www.kidshelp.com.au/)), DirectLine (Phone 1800 888 236), or LifeLine (Phone 13 11 14). Kids Help Line is a free and confidential telephone counselling service for 5 to 25 year olds in Australia. Kids Help Line is Australia's only free, confidential and anonymous helpline available for children and young adults. DirectLine provides 24-hour, 7-day counselling, information and referral. At DirectLine, you can talk to professional counsellors who are experienced in alcohol and drug-related matters. DirectLine is free, anonymous and confidential. LifeLine also provides 24-hour, 7-day counselling, information and referral. It is also staffed by trained professional counsellors and offers mental health and crisis support.

**6. Do I have to take part in this research project?**

Participation in any research project is voluntary. If you do not wish to take part, you do not have to. If you decide to take part and later change your mind, you are free to withdraw from the project at a later stage. If you decide to withdraw, please notify a member of the research team. This notice will allow that person or the research supervisor to inform you if there are any special requirements linked to withdrawing. If you decide to leave the project, the researchers would like to keep the personal and/or health information about you that has been collected. This is to help them make sure that the results of the research can be measured properly. If you do not want them to do this, you must tell them before you withdraw from the research project. Your decision whether to take part or not, or to take part and then withdraw, will not affect your relationship with the researchers or Eastern Health, Deakin University, *drummond street services*, or any other service involved in this research.

7. How will I be informed of the final results of this research project?

The results of this project will be published in several scientific journals. Please email [andrew.lewis@deakin.edu.au](mailto:andrew.lewis@deakin.edu.au) for a summary of the findings or copy of any report. The final results should be available by December 2013.

8. What will happen to information about me?

Any information that we collect for this research study that can identify you will remain confidential. The information we collect will be kept in a locked cabinet at Deakin University for a minimum of 15 years after the results are published. After the 15 years are up, the information will be destroyed in a safe way to protect your privacy.

Your information that is entered into the computer database, and the written and phone surveys that you complete, will be labelled with a number instead of your name to protect your privacy. There will be one copy of the list matching your number with your name, and this will be stored in a locked cabinet at Deakin University along with your consent form, your contact details, and the audio recordings of the groups. Except as required by law, any information that we collect that can identify you will be kept confidential, and destroyed safely when it is no longer required. Only members of the research team will have access to your files.

In any publication or presentation of the findings, we will present the information in such a way that you cannot be identified, unless you are asked first and agree to it in writing.

9. Can I access research information kept about me?

## *Yes. The privacy and other relevant laws in Australia and/or Victoria state that you have the right to access the information collected and stored by the researchers about you. Please contact one of the researchers named at the end of this document if you would like to access your information. The information collected in this research project will be kept for at least 15 years, in accordance with regulations.*

10. Is this research project approved?

## *The ethical aspects of this research project have been approved by the Human Research Ethics Committees of Deakin University and Eastern Health. This project will be carried out according to the National Statement on Ethical Conduct in Human Research (2007) produced by the National Health and Medical Research Council of Australia. This is a statement that was created to protect people who agree to participate in human research studies.*

11. Who can I contact?

If you want to contact someone about any part of this study, you can work out who might be best to contact by reading the next section. **For further information or appointments:**

If you want any further information about this project or if you have any problems from being in the project (for example, feeling very worried or upset), you can contact this person:

Name: Associate Professor Andrew Lewis

Position: Principle Researcher

Telephone: 92546774

Otherwise, you can contact any of the following people who are also researchers on this project: John Toumbourou 52278278, Tess Knight 92546595, or Melanie Bertino 92517364.

If you need help but you can’t reach us, you can also try contacting your local doctor if they are available, or you can phone one of the telephone counselling services previously described (Kids Help Line (Phone [1800 55 1800](http://www.kidshelp.com.au/)), DirectLine (Phone 1800 888 236), or LifeLine (Phone 13 11 14)).

**For complaints:**

If you have any complaints about any part of the project, the way it is being run or any questions about being a research participant in any study, then you can contact:

Ethics Chairperson, Eastern Health Human Research and Ethics Committee, Phone 03 9895 3398, Email ethics@easternhealth.org.au

Consent Form for Youth (aged 12-18)

FILL OUT THE TOP SECTION OF THIS FORM IF YOU WANT TO BE IN THE STUDY

If you want to be in this study, please fill out your name, your parent’s name, sign and date below, and send it back.

I have read this form, or else I have had this form read to me, in a language that I understand. I understand the information in this form about the research study. This includes the reason for the study, the things that I and others will be asked to do if I choose to take part in this study, and the possible benefits and risks of the study.

The researcher has asked me if I have any questions and has answered any of my questions properly. I freely agree to participate in the research project that is described in this form.

I agree to the audio recording of my telephone interviews, and of the group treatment sessions, should I choose to go along to the groups with my parents. I understand that these tapes of the sessions and telephone interviews will only be accessed by research team members, will not be copied or distributed, and will be kept in a secure locked location.

I understand that the researchers have agreed not to reveal my own or my family’s identity and personal details without my consent, unless if required by law.

I understand that I will be given a signed copy of this document to keep.

My name (Participant) (printed) ………………………………………………………………

My parent’s name/s (printed) …………………………………………………………………

My Signature ……………………………………………………… Date

Declaration by researcher*: I have given a full written explanation of the research project, its procedures and risks and I believe that the participant has understood that explanation.

Researcher’s name (printed) ………………………………………………………………………………..

Signature ……………………………………………………… Date

*Note: All parties signing the consent section must date their own signature.*

OR, FILL OUT THIS SECTION OF THE FORM IF YOU DO NOT WANT TO BE IN THE STUDY

If you do not want to be in this study, please only fill out your name and address below, and send it back. Please do not fill out or sign the above part of this form.

I do not want to take part in this study. Please send my $15 voucher for sending this form back, to the address I have written below. I understand that this means that I or my family will not be involved in this research study.

Name:

Address:

**PARTICIPANT INFORMATION and consent form (PICF) For SIBLINGS OF ADOLESCENTS/YOUNG ADULTS (12-18)**

Participant Information and Consent Form: For multiple Eastern Health sites, including Turning Point Alcohol and Other Drug Services and CYMHS; Deakin University; Headspace Collingwood; and for *drummond street services*

Date: January 2012

**Full Project Title:** Engaging youth with high prevalence mental health problems using family based interventions

**Brief Project Title:** Family Options Study

**Principal Researcher:** A/Prof Andrew Lewis

**Associate Researchers:** Prof John Toumbourou, Dr Tess Knight, Dr Melanie Bertino, Prof Dan Lubman, Dr Steven Leicester

**Student Researchers:** Julian Thompson, Ai Tran, Joanna Skewes, Milanda Matthews and Jaclyn Danaher

This Plain Language Statement and Consent Form is 8 pages long. Please make sure you have all the pages.

1. Introduction

You are invited to take part in a research study, because your brother or sister has agreed to you taking part in a group with them and your parent/s. This study invites parents and young people to come along to a group program once a week for 2 hours with other families. If you chose to participate in this research you can join in the group with your parents and sibling, but you do not have to if you don’t want to. This research study aims to compare how parents and young people are feeling both before and after their family has participated in the counselling group.

This Participant Information and Consent Form tell you about the research project. It explains what is involved to help you decide if you want to take part in this study. *Please read this Plain Language Statement carefully.* Feel free to ask questions about any information in the document. You may also wish to discuss the project with a relative or friend or your local health worker. Feel free to do this. Once you understand what the project is about and if you agree to take part in it, you will be asked to sign the Consent Form. By signing the Consent Form, you indicate that you understand the information and that you give your consent to participate in the research project.

1. **What is the purpose of this research project?**

The purpose of this project is to examine the effectiveness of two counselling groups to find out if working with the family in these counselling groups can help to reduce young people’s depression or other mental health problems. Another aim of the study is to find out if the groups can help young people to get connected with a mental health service should they want to. We are also looking at studying whether the counselling groups can help parents to have better mental health. This is important research because it will help us to better understand what treatments work best for parents and young people with these mental health concerns. This is likely to lead to better care for other families in the future.

Research suggests that approximately 80 percent of young people with a mental illness do not receive any suitable treatments, and that many young people are reluctant to attend a treatment for their mental health problems. A few studies have shown that working with the families of youth with depression or drug and alcohol problems can have a positive impact on young people and other family members. However more studies are needed about this. Also very few or no studies have been done about whether young people are more likely to attend a treatment for depression after their parents have attended a counselling group first.

Overall, we hope that 180 families will take part in this research study over the next two years. Most of these families (up to 145 families) will take part at one of the Eastern Health or Deakin sites, and the rest (about 35 families) will take part at the drummond street services site. There are two different groups in the study. Each group is designed for parents/families of youth aged 12-18. Both groups provide support and education to parents. During both groups young people will be invited to join in at a later stage. The project is only running in Melbourne and Geelong, Australia at the sites listed on this form.

This research study builds on earlier research done by a team of people at Deakin University and drummond street services in partnership with beyondblue: the national depression initiative. The earlier project compared a one-to-one youth treatment with a family group for youth depression. The group treatment was well received and helped young people and their families to feel better overall than before the group began. Four of the researchers from this earlier project are also working on this study. Two new researchers have also joined the team, named Professor Dan Lubman and Dr Steven Leicester from Eastern Health. This research has been funded by: beyondblue, the Australian Drug Foundation, drummond street services, Deakin University and the Australian Research Council.

The following people are student researchers who will be working on the project: Julian Thompson, Ai Tran, and Jaclyn Danaher and others yet to be confirmed. These students are postgraduate students completing the professional masters or doctoral program in psychology at Deakin University. The students will assist the project by helping with assessments, groups, and data, and writing up parts of the research outcomes for their thesis which will count towards their academic record.

This research has been funded by: *beyondblue,* the Australian Drug Foundation, *drummond street services,* Deakin University and a grant from the Australian Research Council.

1. **What does participation in this research project involve?**

**Procedures**

Participation in this project will involve filling out a questionnaire at three separate times: at the beginning of the study; at the end of the groups; and 6 months after the treatment. Should you chose to discontinue in the study for any reason, you will also be given the option to provide feedback on your reasons for opting out. Should your family chose to go on a waiting list for treatment as part of the study rather than taking a different service option, you may be invited to complete another questionnaire prior to starting the treatment for which you have waited. A ‘waiting list’ refers to a period of time whereby there are no clinicians or times available for treatment, and thus families are given the option of waiting for treatment until there is an available time and place for them to attend.

If you chose to take part in this project the following steps will happen:

1. Your parent or guardian will tell us some information about you and your family over the phone.
2. We will talk to your sibling who may be experiencing depressive symptoms.
3. If the researchers are very worried about anyone in your family because of what you, your sibling or your parents tell us, they will work with the members of your family to develop a safety plan to try to keep everyone safe.
4. If your family is eligible, then we will send you a consent form (included with this form) and some questionnaires. We will ask you to fill in the last page of this form, and sign it, and also to fill in the questionnaires, and then return it all to us by post. Your parent/s and sibling will also be asked to complete their own consent form and questionnaires. If you would like assistance completing these surveys, the researchers can arrange for you to visit the university, or for us to visit your home to assist you. Then, your family will be invited to go into a group in this research project.
5. Your family will be invited to go into a group in this study. When the group is full your parents will be told the time and date when it will start.
6. We will send you and your parent(s) some written surveys to complete, and send them back to us.
7. We will invite you to complete another written survey, two more times – at the end of the group and six months after the group.

The written surveys ask about your general and mental health (such as your moods and feelings, your activities, and your sleeping patterns, and recent drug use). Each survey booklet should take about 25 minutes for you to complete. You will receive a $10 Coles-Myer, JB Hi-Fi or iTunes voucher for each completed booklet. The information from your written surveys will be given a code, and will be securely stored in your research file.

Once you have completed the written survey and we have received your completed consent form, your parent/s and sibling will also need to return their surveys and then they will be put into a group. When the group is full your parents will be told the time and date when it will start. The groups will probably run on a weeknight in the early evening. The groups will run weekly for 8 weeks during the school terms, for 2 hours each time. Your will be invited to attend the group with your parent/s after about 4 weeks if you want to, but you do not have to if you don’t want to*.*

If you decide not to do any of these things listed above, it will not in any way affect your relationship or your parents’ relationship with any of the people or agencies that are running this study.

1. **Groups**

The groups will probably run on a weeknight in the early evening. The groups will run weekly for 8 weeks during the school terms, for 2 hours each time. You and your sibling will be invited to attend the group with your parent/s, and your involvement is voluntary*.*

There are two different groups running as part of this study, and your parents will be put into one of the groups if you decide to take part. We think both groups will be helpful for parents and families. We are studying them to find out if one group is more helpful than the other, or if they are the same. Families will not get to choose which group they go to, this will be allocated by the researchers in a random order. Putting people in to groups in a random order helps the researchers to make sure that the final results of the study are more accurate and not influenced or changed in unscientific ways by the people doing the research.

1. Privacy, Confidentiality and Disclosure of Information

Your personal information that you give to the research team will be kept in two a secure and locked file (including your personal details, consent form, and your survey responses. If you want to access your personal information, the research team can help you to do that.

- Your information will be kept private unless you want the researchers to give your private information to another person (such as your parent/s). If you do want the research team to give another person your information, you must ask for this in a letter or email and send it to the researchers. Sometimes in rare cases, some of your private information may also be released without your approval, but only if it was required by law or of there was an ethical obligation (e.g. to protect you or other people from coming to serious harm) – see section 8 of this form for more information.

You can be assured that you and your family will not be identified by name in any way in the reporting of our results. The information we collect will be stored in a locked cabinet within the School of Psychology at Deakin University for a minimum of 10 years from the date of publication. Any information obtained in connection with this project and that can identify you will remain confidential. It will only be disclosed with your permission, subject to legal requirements.

It is also important to understand that the group sessions will be audio-taped. This will allow the research team to see how well the counselors have run the groups as they are supposed to be run. These recordings will only be listened to by members of the research team. Recordings will be kept in a secure place, and will not be shared with anyone outside of the research team. If you participate in this study, your written and phone survey responses will also be kept in a locked place at Deakin University. All of these things will be safely destroyed after 15 years.

1. **Reimbursement**

The group sessions will be provided to your family free of charge. You will not be paid for your participation in this research, but you will be reimbursed for your time spent on this research. The written questionnaire will ask about your general and mental health, such as your moods and feelings, your activities, and recent drug use. Each questionnaire should take about 30 minutes for you to complete. For every questionnaire completed, you will receive a $10 Coles-Myer, iTunes or JB-HiFi voucher of your choosing, which will be $45 in total if you complete all 3 of them. The information from your phone interview and your written surveys will be given a code, and will be stored securely in your file.

1. **What are the possible benefits?**

Possible benefits of being in this research include receiving free mental health services (including assessment and treatment), including a program that is likely to improve your parent’s and/or your siblings mental health and reduce stress, and which may also improve your mental health. However, we cannot guarantee or promise that you and your parent/s or siblings will receive any benefits from this project. This study is also important for knowing how to best help other families in the future.

1. What are the possible risks?

### *If you tell the researchers the details or facts about an illegal activity, they cannot promise to keep this secret from the authorities. So you should not give any specific details about crimes or illegal activities at any time to the researchers, including during the groups (e.g. who was there, what happened, where it happened). If you do, this could be used against you or your family.*

### *It is not anticipated that you will experience any risk or side effects directly from being involved in this study as you will be in a supportive environment and receiving treatment. Your family will be in a supportive environment and receiving a treatment group. Should you experience discomfort during the treatment process and wish to seek independent counselling please contact your local doctor. You may also call Kids Help Line 1800 55 1800, DirectLine 1800 888 236 or LifeLine 13 11 14*

**Kids Help Line** is a free and confidential, telephone counselling service for 5 to 25 year olds in Australia. Kids Help Line is Australia's only free, confidential and anonymous helpline available for children and young adults.

**DirectLine** provides 24-hour, 7-day counselling, information and referral. At DirectLine, you can talk to professional counsellors who are experienced in alcohol and drug-related matters. DirectLine is free, anonymous and confidential.

**LifeLine** also provides 24-hour, 7-day counselling, information and referral. It is also staffed by trained professional counsellors and offers mental health and crisis support.

You are free to withdraw from the study at any time. Please note that if you choose to withdraw from the study, this will not lead to your brother/sister or parent/s being removed from the program as well.

If you become upset or bothered because of the research, the researcher can help you to find other counselling or other support if you want it. Any other counselling or support can be provided by different staffs who are not members of the research team.

1. **Do I have to take part in this research project?**

Participation in any research project is voluntary. If you do not wish to take part, you do not have to. If you decide to take part and later change your mind, you are free to withdraw from the project at a later stage. If you decide to withdraw, please notify a member of the research team. This notice will allow that person or the research supervisor to inform you if there are any special requirements linked to withdrawing. If you decide to leave the project, the researchers would like to keep the personal and/or health information about you that has been collected. This is to help them make sure that the results of the research can be measured properly. If you do not want them to do this, you must tell them before you withdraw from the research project. If you decide not to do any of these things listed above, it will not in any way affect your relationship or your parents’ relationship with any of the people or agencies that are running this study. If you decide not to do any of these things listed above, it will not in any way affect your relationship or your parents’ relationship with any of the people or agencies that are running this study.

1. **How will I be informed of the final results of this research project?**

The results of this project will be published in several scientific journals. Please email [andrew.lewis@deakin.edu.au](mailto:andrew.lewis@deakin.edu.au) for a summary of the findings or copy of any report. The final results should be available by December 2013.

1. What will happen to information about me?

Any information that we collect for this research study that can identify you will remain confidential. The information we collect will be kept in a locked cabinet at Deakin University for a minimum of 15 years after the results are published. After the 15 years are up, the information will be destroyed in a safe way to protect your privacy.

Your information that is entered into the computer database, and the written and phone surveys that you complete, will be labelled with a number instead of your name to protect your privacy. There will be one copy of the list matching your number with your name, and this will be stored in a locked cabinet at Deakin University along with your consent form, your contact details, and the audio recordings of the groups. Except as required by law, any information that we collect that can identify you will be kept confidential, and destroyed safely when it is no longer required. Only members of the research team will have access to your files.

In any publication or presentation of the findings, we will present the information in such a way that you cannot be identified, unless you are asked first and agree to it in writing.

1. Can I access research information kept about me?

## *Yes. The privacy and other relevant laws in Australia and/or Victoria state that you have the right to access the information collected and stored by the researchers about you. Please contact one of the researchers named at the end of this document if you would like to access your information. The information collected in this research project will be kept for at least 15 years, in accordance with regulations.*

1. Is this research project approved?

## *The ethical aspects of this research project have been approved by the Human Research Ethics Committees of Deakin University and Eastern Health. This project will be carried out according to the National Statement on Ethical Conduct in Human Research (2007) produced by the National Health and Medical Research Council of Australia. This is a statement that was created to protect people who agree to participate in human research studies.*

14. Who can I contact?

If you want to contact someone about any part of this study, you can work out who might be best to contact by reading the next section. **For further information or appointments:**

If you want any further information about this project or if you have any problems from being in the project (for example, feeling very worried or upset), you can contact this person:

Name: Associate Professor Andrew Lewis

Position: Principle Researcher

Telephone: 92546774

Otherwise, you can contact any of the following people who are also researchers on this project: John Toumbourou 52278278, Tess Knight 92546595, or Melanie Bertino 92517364.

If you need help but you can’t reach us, you can also try contacting your local doctor if they are available, or you can phone one of the telephone counselling services previously described (Kids Help Line (Phone [1800 55 1800](http://www.kidshelp.com.au/)), DirectLine (Phone 1800 888 236), or LifeLine (Phone 13 11 14).

**For complaints:**

If you have any complaints about any part of the project, the way it is being run or any questions about being a research participant in any study, then you can contact:

Ethics Chairperson, Eastern Health Human Research and Ethics Committee, Phone 03 9895 3398, Email: ethics@easternhealth.org.au

Consent Form for Siblings of Youth (aged 12+)

FILL OUT THE TOP SECTION OF THIS FORM IF YOU WANT TO BE IN THE STUDY

If you want to be in this study, please fill out your name, your parent’s name, sign and date below, and send it back.

I have read this form, or else I have had this form read to me, in a language that I understand. I understand the information in this form about the research study. This includes the reason for the study, the things that I and others will be asked to do if I choose to take part in this study, and the possible benefits and risks of the study.

The researcher has asked me if I have any questions and has answered any of my questions properly. I freely agree to participate in the research project that is described in this form.

I agree to the audio recording of the group treatment sessions, should I choose to go along to them with my parents. I understand that these tapes of the sessions will only be accessed by research team members, will not be copied or distributed, and will be kept in a secure locked location.

I understand that the researchers have agreed not to reveal my own or my family’s identity and personal details without my consent, unless if required by law.

I understand that I will be given a signed copy of this document to keep.

My name (Participant) (printed) ………………………………………………………………

My Signature ………………………………………………………………… Date

My parent’s name/s (printed) …………………………………………………………………

My Parent’s Signature ………………………………………………………… Date

Declaration by researcher*: I have given a written explanation of the research project, its procedures and risks and I believe that the participant has understood that explanation. I have also verbally explained the procedures and risks of this research project to this participant’s parent or guardian.

Researcher’s name (printed) ………………………………………………………………………………..

Signature ……………………………………………………… Date

*Note: All parties signing the consent section must date their own signature.*
